# Large enhancement of simultaneous color contrast by white flanking contours

**DOI:** 10.1038/s41598-020-77241-5

**Published:** 2020-11-18

**Authors:** Tama Kanematsu, Kowa Koida

**Affiliations:** 1 Department of Computer Science and Engineering, Toyohashi University of Technology, Toyohashi, Aichi 441-8580 Japan; 2 Research Fellow of Japan Society for the Promotion of Science, Tokyo, Japan; 3 Electronics-Inspired Interdisciplinary Research Institute (EIIRIS), Toyohashi University of Technology, Toyohashi, Aichi 441-8580 Japan

**Keywords:** Colour vision, Human behaviour, Perception

## Abstract

Simultaneous color contrast and assimilation are mutually opposing effects on color appearance, and their magnitude depends on spatial context. The Monnier–Shevell illusion induces a large color shift by a synergy of simultaneous assimilation and contrast using the alternating color of proximal and distant surrounds. The illusion induces a prominent effect along the blue-yellow color axis, but a subtle effect along the orthogonal color axis. In this study, we report an illusion generated by an extremely thin gray line on a cyan background that appears reddish when the line is flanked by thin white contours. We quantified the color appearance of the gray line in a color matching experiment and found that the color shift of the gray line with white contours induced large color shifts. It is also known that luminance contrast between a center and its surrounds affects the magnitude of simultaneous color contrast. However, our color contrast effects were larger for a dark line rather than for a pale line. In contrast, the perceived color shift of the line without the contours increased as the luminance of the gray line increased, supporting the known effect of Kirschmann’s third law. These results indicate that Kirschmann’s third law fails to explain the perceived color shift of our illusion, even after accounting for optical factors like aberrations. Observed color shifts could be explained by an augmented synergy theory based on intensity space, rather than chromaticity.

## Introduction

**Figure 1:**
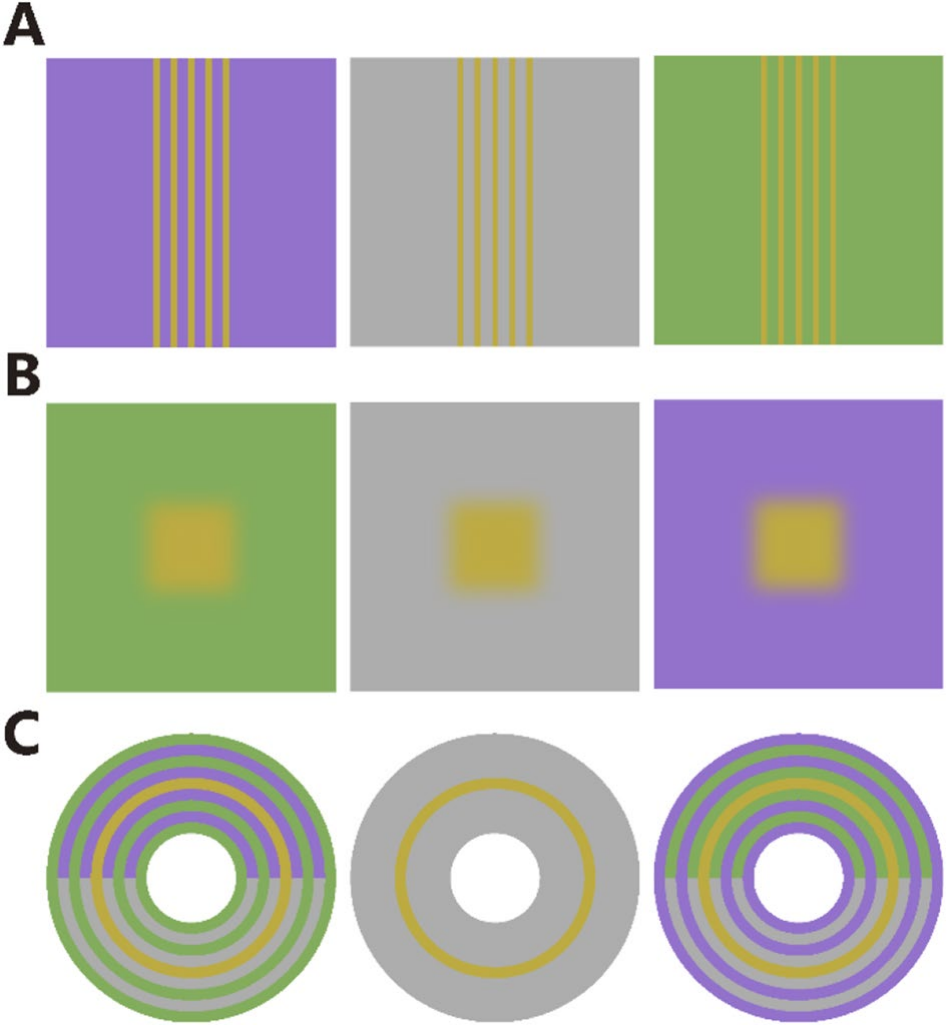
Effect of surrounds on color appearance. (**A**) Simultaneous color assimilation. The thin orange bars on the left appear purplish, whereas on the right they appear greenish. The bars appear similar to the color of each background. (**B**) Simultaneous color contrast. The orange square on the left appears purplish, whereas on the right it appears greenish. The squares appear similar to a complementary color of each background. (**C**) Monnier–Shevell illusion^25,27^. The orange ring on the left appears purplish, whereas on the right it appears greenish. Illusory color emerges by the synergy of the assimilation effect from a proximal ring and the contrast effect from a distant ring. The illusion is valid whether the proximal ring is colored (top) or neutral (bottom). In the center column, the orange bars, square, and ring are surrounded by a gray background for reference.

Color appearance depends on spatial context. Simultaneous color assimilation induces color appearance from the surround towards a central area, whereby the color appearance is biased towards the color of the surround (Fig. 1A). This contributes to spatial averaging for more reliable color signal detection. In contradistinction, simultaneous color contrast induces the appearance of the complementary color from the surround towards a central area, whereby the color appearance is biased towards a color opposing the color of the surround (Fig. 1B). This process supports the detection of low contrast objects and is a contributing factor in color constancy^1,2^. These contextual effects emerge for any hue composition of the surround^3^.

Simultaneous color assimilation and contrast depend on the spatial configuration of luminance variations in an image. Color assimilation becomes larger when the luminance contrast between the center and the surround area is high^4–7^. Wavelength-dependent and wavelength-independent optical factors do not fully account for color assimilation, so it is believed that neural factors mainly contribute to this effect^4^. However, the magnitude of the simultaneous color contrast is optimized when the luminance contrast between the surrounds and the center is absent, and this effect is greatly attenuated by the luminance contrast, known as Kirschmann's third law^8^. The law has been examined repeatedly by various methods of scaling^9^, naming^10^, rating^11,12^, matching^13,14^, and canceling^15^ to measure the perceived change in color, with the majority of findings from these studies supporting the law. The variation in color appearance depending on the stimulus luminance has long been investigated in the context of color constancy^16–24^. Color constancy is a feature that assigns constant colors to objects under varying illumination conditions. However, under colored illumination, a discrepancy remains between Kirschmann’s law and the dependence of color appearance on luminance. A darker target is susceptible to the surrounds; this is known as the Helson–Judd effect^16–24^.

Simultaneous color assimilation and contrast also depend on the spatial frequency of the spatial extent. Color assimilation tends to be perceived in high spatial frequency stimuli^3,4^, while color contrast tends to be perceived in relatively low spatial frequency stimuli^3^. In other words, color assimilation is perceived from proximal surrounds, and color contrast is perceived from distal surrounds.

Using the alternating color of proximal and distal surrounds, Monnier and Shevell reported an illusion inducing a large color shift by the synergy of simultaneous color assimilation and contrast (Fig. 1C, top). The magnitude of the color shift is large even when the proximal surrounds have a neutral color (Fig. 1C, bottom) compared to the color shift of uniformly colored surrounds^25^ (Fig. 1B). These color shifts were explained by receptive field organization in which an additive effect arising from a proximal surround and a subtractive effect arising from a distal surround^25,26^. This illusion reflects not only the synergistic effect, but also the luminance contrast of the center^7^. The greater the luminance contrast of the center, the larger color shifts that occur.

The Monnier–Shevell illusion was reported as color shifts occurring along the short-wavelength cone (S cone) direction^27^. There were reports showing the same illusions along red-cyan colors (long to medium wavelength cone direction; L–M cone), but the magnitude of these effects was smaller^7,28^. Considering that classical color assimilation and contrast emerges across all hues^3^, it is questionable that synergistic color shifts are constrained along the S cone axis. If we find conditions that produce a strong color shift that are oblique to the S cone direction in color space, then the proposed explanation would be generalizable to more than one independent color axis. This potential generic explanation is simpler than the complex explanation requiring a different function for each of the cone axes. The simple explanation would help to understand human visual processing.

**Figure 2:**
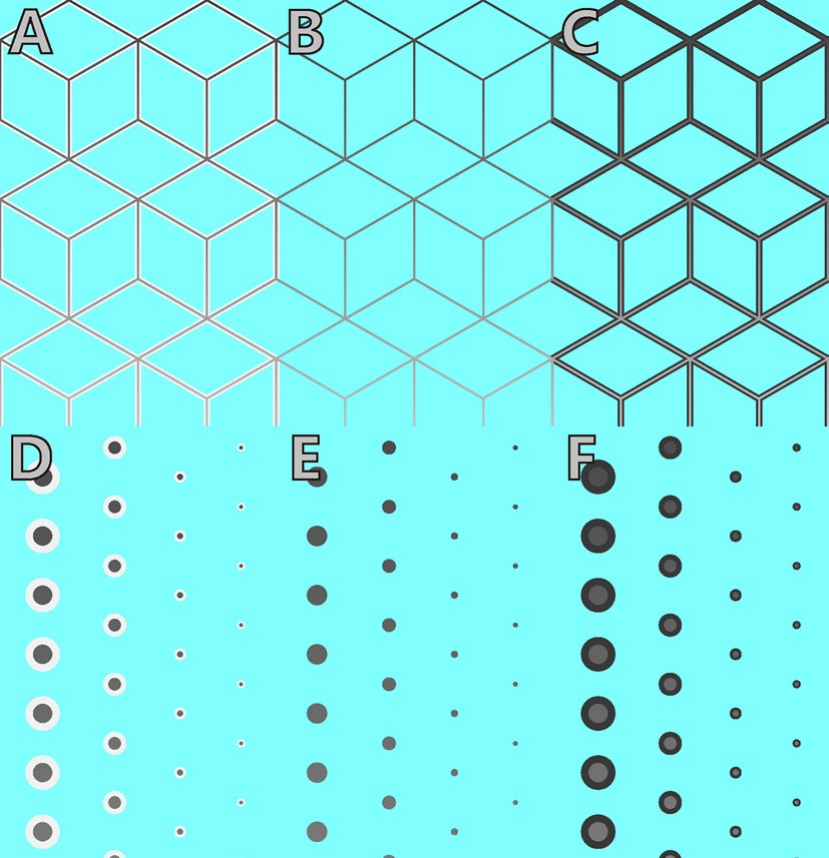
Simultaneous color contrast effects were robustly induced when gray lines were flanked by white contours. (**A**) Color shifts were obvious when the gray lines were flanked by white contours on the cyan background, and the gray lines appeared reddish. Adjust your visual distance and the stimulus size for better effects. (**B**) The effect was weak without white contours. (**C**) Color shifts were almost absent when the gray lines were flanked by black contours. (**D**) The spot pattern also induced illusory effects. The size of the gray spots with white contours decreased from left to right. The effect was prominent for the small spots. (**E**) Weak effect for the spots without white contours. (**F**) Absent effect for the spots with black contours. The luminance of the gray lines and spots gradually increased from top to bottom.

Although a synergistic effect along the S cone axis and its dependence on luminance contrast is already known to exist, it is unknown whether this synergistic effect persists along red-cyan color axis and whether it is influenced by luminance contrast. In this study, we examined whether color appearance varies depending on the existence of a contour. We report the finding of an illusion that induces strong contrast effects from a distal area along the red-cyan color axis. Gray lines flanked by white contours were found to appear red when viewed against a (complimentary) cyan background (Fig. 2A). However, gray lines without flanking contours appeared hardly red (Fig. 2B). The effect was practically absent when gray lines were flanked by black contours (Fig. 2C). The effect was not limited to the presentation of lines, but also applied to small spots (Fig. 2D–F) and still evident when both the gray area and the white contours were thin or small. The effect was robust against changes in the orientation of the lines, across visual fields, and display devices used for presentation. Below, we compared the magnitude of the color shifts between different contrasts of the contour to test whether the spatial explanation of the color induction from proximal and distal surrounds is valid or not, irrespective of the luminance contrast of the proximal surround. We finally measured the effect of the luminance level of the gray line. If the luminance dependency is similar to that observed for the S cone axis, then high contrast stimuli would induce larger color shifts.

## Results

### Appearance matching

**Figure 3:**
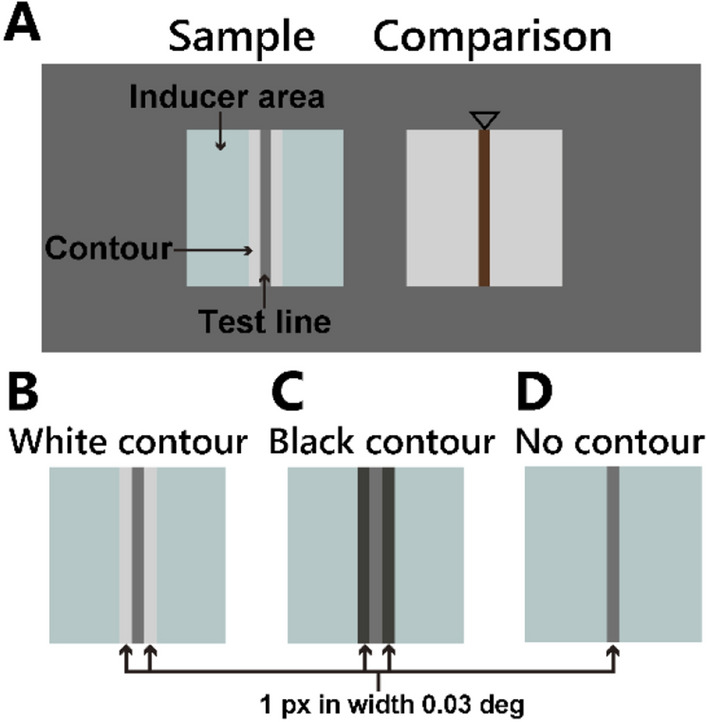
Stimuli used in the appearance matching experiment. (**A**) The sample and comparison stimuli sized at 3.12° arranged horizontally and separated by 1.24° on a uniform gray screen. (**B**) Sample stimuli of the white-contour condition. A gray vertical line (test line) was placed on a uniform colored background (inducer area). White lines (white contour) were located adjacent to both sides of the test line. (**C**) Sample stimuli of the black-contour condition. Black lines (black contour) were located adjacent to both sides of the test line. (**D**) Sample stimuli of the no-contour condition. The test line was placed on the inducer area without any contour. The width of both the contour and the test line was 1 px (0.03°).

We quantified the appearance of the gray line using an asymmetric color-matching experiment. Five participants observed a sample line and a comparison line stimulus. They adjusted the pixel values of the comparison's line to match the appearance to the sample's line (Fig. 3A). The adjustment was performed two dimensionally using a trackball, with one axis of rotation modifying color from red to cyan and the other axis of rotation modifying luminance.

The sample's line was a D65 gray vertical line (*test line*) placed in the center of a chromatic surround area (*inducer area*). The sample had three conditions; (1) *white-contour condition*, in which white lines flanked the test line (Fig. 3B), (2) *black-contour condition*, in which black lines flanked the test line (Fig. 3C), and (3) *no-contour condition*, in which no lines flanked the test line (Fig. 3D). The test line had three levels of luminance to test Kirschmann’s law (see “Materials and Methods” for details).

**Figure 4:**
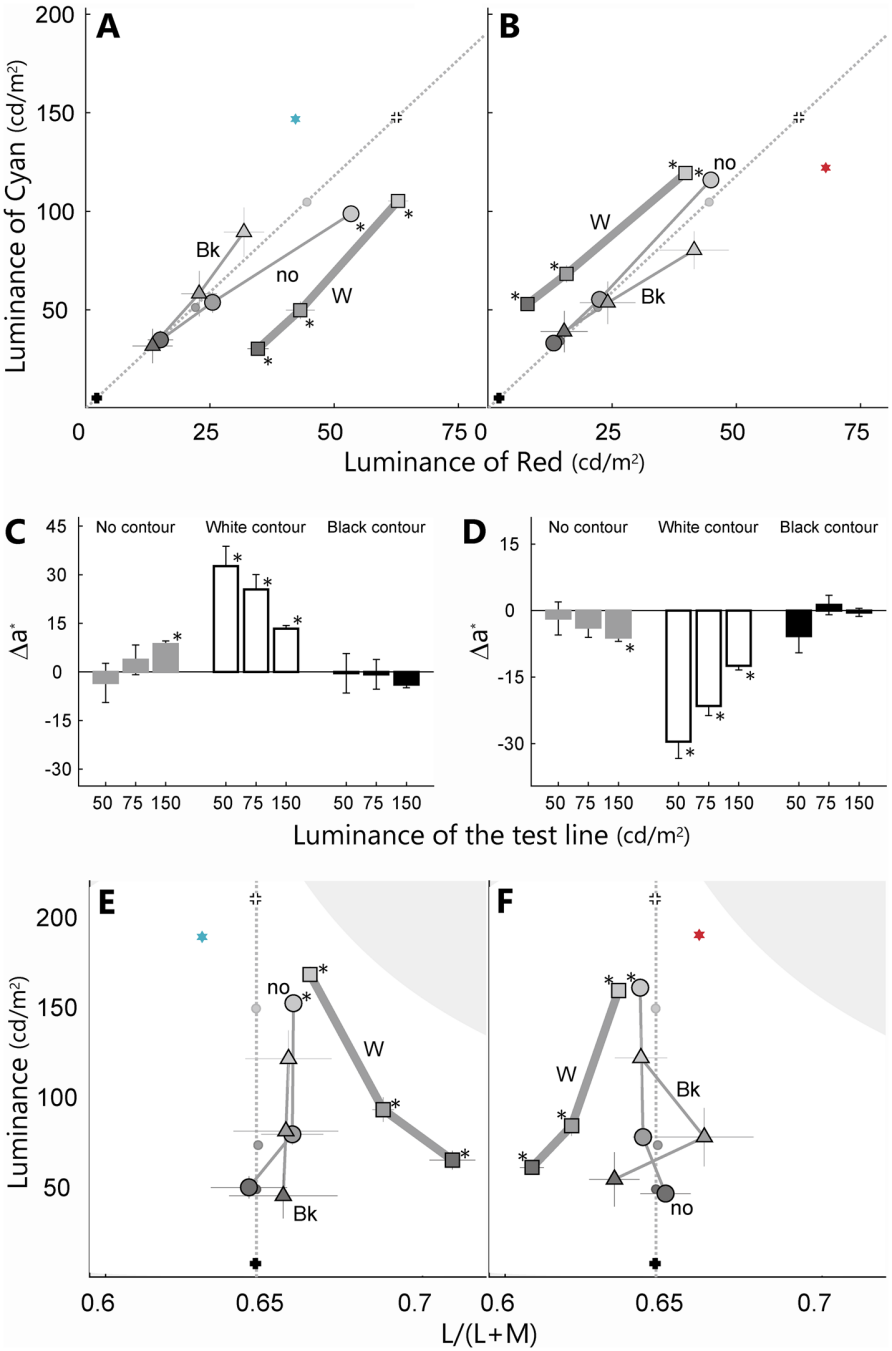
Results of the appearance matching averaged across all participants (n = 5). The panels show the results of the cyan (**A**,**C**,**E**) and red inducers (**B**,**D**,**F**). (**A**) and (**B**) are scatterplots describing the color and luminance of the stimuli. One axis is the luminance of the red phosphor and the other axis is the luminance of the cyan (green + blue) phosphor. The diagonal line represents neutral conditions (D65). Small dots on the diagonal line represent the three levels of the test lines. The colored star represents the inducer of the sample. Circles, squares, and triangles represent the matched color of the no-contour, the white-contour, and the black-contour conditions, respectively. White and black crosses represent the color of the white and black contours of the sample stimuli, respectively. (**C**) and (**D**) summarize the color shift quantified by ∆a* of the CIELAB color space. A positive value on the vertical axis indicates a reddish, a negative value indicates a cyanic, and zero indicates no color shift. The horizontal axis shows the luminance of the test line. Gray, white, and black bars show the results of the no-, white-, and black-contour conditions, respectively. (**E**) and (**F**) summarize the color shift quantified by cone excitation space. The horizontal axis is relative cone excitation of L-cone divided by sum of L- and M-cone, and the vertical axis is luminance. The markers are same as in (**A**). Gray area denotes outside of the display gamut. The error bar is the standard error of the mean. An asterisk indicates a significant difference between each matching and a neutral gray color [p < 0.05, sign test in (**A**), (**B**), (**E**), (**F**), t-test in (**C**), (**D**)].

The results showed that the color appearance differed between stimulus conditions. For the no-contour condition of the cyan inducer, the matched color of the pale test line significantly shifted toward the opposite color from cyan (circle markers in Fig. 4A, sign test; p < 0.05). In contrast, no significant shift was observed for the other two test lines. This indicates that the magnitude of the simultaneous color contrast effect increased as the luminance contrast between the test line and the inducer area decreased, supporting Kirschmann’s law.

For the white-contour condition of the cyan inducer, the matched color significantly shifted toward the opposite direction from the inducer color irrespective of the luminance of the test line (square markers in Fig. 4A, sign test; p < 0.05 for three luminance conditions). These shifts indicate simultaneous color contrast. For the black-contour condition, the matched colors did not significantly shift from neutral (triangle markers in Fig. 4A, sign test; p > 0.05). These trends were replicated for the red inducer (Fig. 4B). The white-contour condition induced a contrast effect (sign test; p < 0.05 for all three luminance conditions).

The observed color shifts were transformed into the CIELAB color space, which is an approximate uniform color scale describing perceptual color differences (Fig. 4C,D). Because the distance on the luminance axes in Fig. 4A,B are not indicative of perceptual color differences, the uniform color scale was used to compare the perceived color shifts between stimuli of different luminance levels. One of the color axes, ∆a*, of the CIELAB space corresponded to the color difference along the red to cyan axis for our stimuli.

Again, the no-contour condition induced a moderate color contrast effect. A significant shift was observed only for the pale test (t-test; p < 0.05). This effect may support Kirschmann’s third law, but the effect of luminance for the test line was not significant (cyan; p > 0.05, F(2, 42) = 1.85, red; p > 0.05, F(2, 42) = 0.70, 1-way repeated measures ANOVA). The white-contour condition induced a significant color contrast effect for all luminance levels (t-test; p < 0.05), and its magnitude was significantly larger than that for the no-contour condition (p < 0.05, t-test between the white-contour and the no-contour condition for each luminance level). Their magnitudes decreased as the luminance of the test line increased (cyan; p < 0.01, F(2, 42) = 27.18, red; p < 0.05, F(2, 42) = 15.87, 1-way repeated measures ANOVA), contrary to the prediction based on Kirschmann's law. The black-contour condition induced no significant color shifts (t-test; p > 0.05).

The observed color shifts were again transformed into the luminance and cone excitation chromaticity (L/(L + M)) space (Fig. 4E, F), which is widely used in color research. The white-contour condition again induced significant color shifts (sign-test; p < 0.05 for three luminance conditions). Significant color shifts of the no-contour condition were observed only for the pale test (sign test; p < 0.05). The black-contour condition induced no significant color shifts (sign test; p > 0.05). The result of the black contour may not appear similar to the result in CIELAB space, because some observers matched to low luminance appeared black, irrespective of color and sometimes resulted in very large values for L/(L + M). Thus, results showed a large deviation in color settings (see the Supplementary Figs. S1 and S2 for individual data).

## Discussion

We provide evidence for a new color illusion that induces large enhancements of simultaneous color contrast using white flanking contours. We quantified the appearance of the thin gray line which was flanked by the white or black contours on a colored background. The white-flanking contour condition induced a large and significant simultaneous color contrast effect, irrespective of the luminance of a centrally flanked gray line. The magnitude of color shift was quantified on CIELAB-∆a*, where we show that the shifts in perceived color decreased as the luminance of the central gray line increased. The color shift for the no-contour condition was significant only for the pale gray line. The black-contour condition induced no significant color shift.

**Figure 5:**
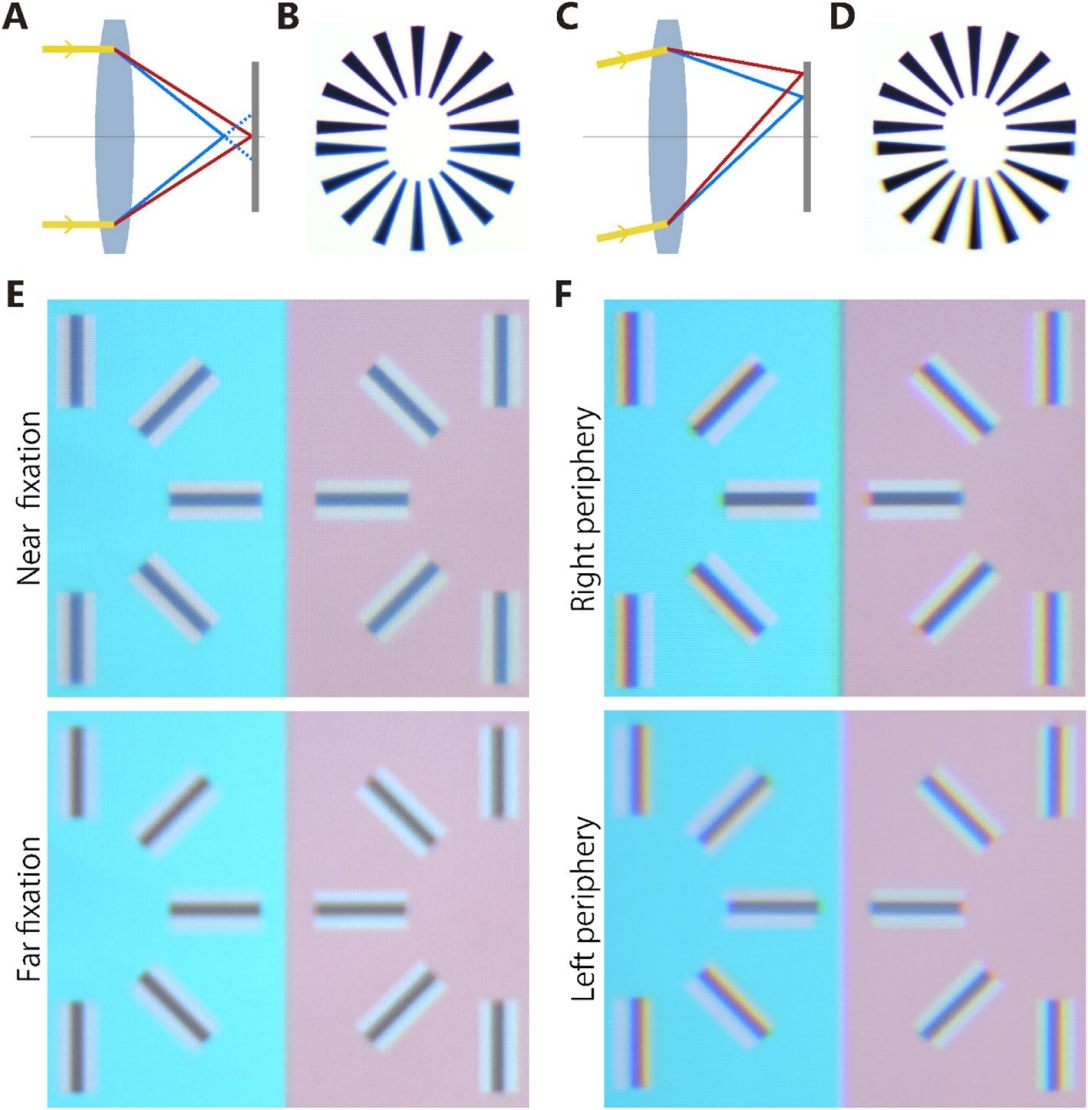
Chromatic aberrations. (**A**) Longitudinal aberration. Short-wavelength light is focused at position nearer to the lens than that of long-wavelength light, thus blurred on the image plane where long-wavelength lights are focused. (**B**) Demonstration pictures of original (top) and a longitudinal aberration case (bottom). Photos were taken by using a non-achromatic lens. In the bottom case, short-wavelength light is blurred and bluish fringe emerges around black lines. The effect is not dependent on the orientation of the lines. (**C**) Lateral aberration. Long-wavelength light is focused at farther peripheral position than that of short-wavelength light. (**D**) Demonstration pictures of the original (top) and a lateral aberration case (bottom) were taken by using a non-achromatic lens. Red and blue fringes emerged on the edge of the black areas. The effect emerges horizontally, thus causing a different outcome depending on the orientation of the edge. (**E**) Effect of longitudinal aberration on the illusion images when the image sensor was placed far from (equivalent to the near fixation depth) and near to (equivalent to the far fixation depth) the lens. The original image presented gray lines flanked by white contours in different orientations on both cyan and red background. On the near fixation image, short wavelength light was blurred and gray lines colored bluish. On the far fixation image, long wavelength light was blurred and gray lines colored reddish. (**F**) Effect of lateral aberration on the illusion images when stimulus presented right (top) and left (bottom) side from the optical axis of the lens. Original images were same as in (**E**). On the right image, the vertical dark lines had colored fringes of bluish on right side and reddish on left side. On the left images, the effect was similar, except that the position of the colored fringe was switched.

Does a wavelength-dependent factor explain the illusion? Chromatic aberration arises from the difference in a refractive index between wavelengths of light. There are two major kinds of chromatic aberrations. First, the longitudinal aberration induces appearance of a colored fringe at edges of the dark and bright parts of the image due to the difference in the focus points along an optical axis (Fig. 5A). At one depth of fixation where a certain wavelength of light is focused on the retina, the other wavelength of light blurs to result in a colored fringe. For example, the black line on the white backgrounds appears bluish at the near fixation depth (Fig. 5B). Longitudinal aberration can be generated in our stimuli, particularly where the gray line is flanked by white contours (Fig. 5E). It should be noted that the outcome is the same between the cyan and red surrounds. In an additional experiment, we examined the color appearance of the gray line for different fixation depths from the monitor. Fixation depth was controlled independently by a discrete fixation annulus shown via a half-transparent mirror (see “Materials and Methods” for details).

At the near fixation, three out of six participants perceived the gray line on the white background as bluish, consistent with what we expected. The three other participants reported the line as appearing achromatic. All six participants perceived the stimuli as reddish when the inducer color was switched to cyan. At the far depth, although no one perceived reddish on the white background, five out of six participants perceived bluish when the inducer color was switched to red. These results indicated that color shifts due to the longitudinal aberrations could occur, but do not exceed the perceptual effect. Thus, longitudinal aberrations do not appear to have been a major contributing factor to the effect.

Second, the lateral aberration caused a color shift tangential to the optical axis, and the short-wavelength light focuses at a near peripheral position (Fig. 5C), resulting in the appearance of a colored fringe at the edge of the lines (Fig. 5D). When a stimulus is placed at right periphery of the visual field, lateral aberrations are evident for vertical lines (Fig. 5D). Lateral aberrations can also be generated by our stimuli (Fig. 5F). It should be noted that the finding was the same between both cyan and red surrounds.

We examined the color appearance of the gray line shown in the right peripheral visual field. Three participants perceived the vertical lines as reddish on the white surround. When the inducer was switched to red, these three perceived the lines as bluish. Although three other participants perceived the white surround as achromatic or green, they perceived it as reddish when the inducer was switched to cyan. Thus, color shifts due to lateral aberration could occur, but did not exceed the size of the perceptual effect. Thus, lateral aberration does not appear to have been a major factor contributing to the illusion.

Color shifts also occur due to wavelength-independent factors such as blurring. Luminance contrasts may change for very thin lines due to the optical blur or neural summation. If image blurring reduces luminance contrast, a strong color contrast effect should be induced as per Kirschmann's law. To test this, we blurred the luminance by averaging the contour and test line. The luminance of the test lines of pale (50 cd/m^2^), middle (75 cd/m^2^), and dark (150 cd/m^2^) then change to be 157, 165, 190 cd/m^2^ for the white contour, 22, 31, 56 cd/m^2^ for the black contour, and 143, 152, 177 cd/m^2^ for no contour, respectively. Thus, the blurred contrast of the center to background will be − 17.5, − 13.2, 0.0% for the white contour, − 88.6, − 84.2, − 71.1% for the black contour, and − 24.6%, − 20.2%, − 7.0% for no-contour condition, respectively. Thus, the lowest contrast is the pale test with white contour. According to Kirschmann’s law, there are two possible predictions. First, the pale test with the white contour (0.0%) should generate the strongest color shift. Second, conditions having similar luminance contrast—i.e., the dark test with the white contour (− 17.5%) and the mid test with the no contour (− 20.2%)—should lead to similar color shifts. These predictions were, however, inconsistent with the results. The magnitude of color shift for the pale test in the white contour condition was not larger than the other two tests in the white contour condition (Fig. 4). The magnitude of the color shift for the dark test with the white contour induced significant color shift, but the mid test with no contour did not. In sum, simple blurring of the test line with Kirschmann's law failed to explain the results.

For the white-contour condition, we observed when the luminance contrast of the test line was higher (darker test line), the color shifts were larger. This trend was similar to recent results. Cerda-company et al. showed that color shifts of the Monnier–Shevell illusion for S-cone stimuli were larger when the luminance contrast of the test was higher. Color shifts along L–M cone stimuli in their study, however, were smaller at all luminance levels, suggesting that a luminance dependency along L–M cone was not observed. This inconsistency could be attributed to the difference in line width between the stimuli.

Monnier and Shevell showed that color shifts were observed only for the S-cone color direction^25,29^, while other studies have found that the effect also occurred along the L–M cone color direction but with smaller illusory effects^7,28^. Our illusion under the white-contour condition using the red-cyan color configuration, was as strong as the Monnier–Shevell illusion. The critical difference between those previous studies using L–M color^7,28^ and our stimuli is the spatial frequency of the stimuli. The typical spatial width used in those studies was wider (0.16°–0.26°) than ours (0.03°)^7,28,30^. It is known that there is a poor spatial resolution for S-cone stimuli (8 cycle per degree, cpd), compared to L–M cone stimuli (20 cpd) and luminance stimuli (50 cpd)^31,32^. There should be a relationship of these differences in the spatial resolution of color vision and illusory effects.

Either synergistically or antagonistically, the effect of proximal and distal surrounds appears to have a simple spatial explanation. This explanation is applicable to our stimuli, but were limited to the white-contour condition. Observed color shifts were small for the black contour condition, indicating the distant contrast effect did not occur. This result of the black-contour condition is consistent with those of previous studies, which reporting that outlining a black line reduced the effect of both assimilation and contrast color^12,33,34^. Thus, the black contour overrides and diminishes both the assimilation and contrast effects and thus, diminishes the synergistic effect.

Observed color shifts of the white-contour condition could be explained by an augmented synergy theory based on intensity space, rather than chromaticity. For the cyan inducer with the white-contour condition, the observed color shifts were almost constant for all three gray levels in luminance space: the shift from the dark test (20.1, − 3.9 cd/m^2^, red and cyan luminance axes respectively), the mid (21.1, − 1.4 cd/m^2^, red and cyan luminance axes respectively) and the pale (18.3, 0.6 cd/m^2^, red and cyan luminance axes respectively). These shifts closely matched the difference between the inducer and the white contour (− 20.3, − 0.8 cd/m^2^, red and cyan luminance axes respectively), except the sign was opposite. For the red inducer, the observed color shifts of the white contour were almost the same among the three gray levels (− 6.6, 18.8 cd/m^2^, − 6.3, 17.0 cd/m^2^, − 4.8, 14.8 cd/m^2^ for the dark, mid and pale line), and the shifts closely matched the difference between the red inducer and the white contour (5.4, − 25.5 cd/m^2^). Thus, contrast effects from distant surrounds based on synergy theory could be regarded as invariant with the changes in luminance of the gray line.

The invariant contrast effect for luminance of the gray line and the applicability of the synergy theory apart from the S cone color are important for explaining the color appearance of our stimuli. Suppose the invariant contrast effect from distal surrounds can be adapted for the no-contour condition, then the synergy theory predicts the observed difference between the white-contour and the no-contour conditions, which is attributed to the proximal assimilation effect. Since the observed difference increased monotonically as the luminance of the gray line was decreased, the color assimilation effect from the proximal surround varied depending on the luminance of the gray line. This dependency is consistent with the former findings^4–7^. The no-contour condition was equivalent to a uniform surround that was widely used in the literature examining the luminance-dependency of simultaneous color contrast effects (Kirschmann’s third law). The luminance dependency could be explained by the augmented synergy theory involving the invariant contrast effect arising from a distal surround and the variable assimilation effect arising from the proximal area.

However, in chromaticity space (Fig. 4C–F), the white-contour condition showed that the darker the gray line, the larger the color contrast effect. This trend is similar to the Helson–Judd effect^16,17^, where the color appearance of a light of fixed chromaticity varies with the intensity under the colored illuminant, and this effect was repeatedly investigated in the context of color constancy^18–24^. The effect was interpreted as the fact that the surrounding color has a large impact on dark targets compared with bright targets^20^.

There is discrepancy between the Helson–Judd effect and Kirschmann’s law. Which effect represents the color contrast effect? They could be reconciled by considering the relative amount of contrast effect to the assimilation effect. Previous studies that investigated the Helson–Judd effect fully adapted to the colored surrounds^16–24^; thus, both spatial and temporal factors enhanced the color contrast effects. The relatively large amount of the contrast effect dominates the color appearance and diminishes the color assimilation effect from the proximal surrounds if it exists. For our white-contour condition, the color assimilation effect from the proximal surround became weak because of the white contour. Thus, a relatively large amount of contrast effect dominated the color appearance. As a consequence, the white-contour condition induced an effect similar to the Helson–Judd effect. We conclude that a consistent feature of the contrast effects is that dark targets are affected more than bright targets, similar to the Helson–Judd effect.

## Materials and methods

All experimental procedures were in accordance with the ethical principles outlined in the Declaration of Helsinki and approved by the Committee for Human Research at the Toyohashi University of Technology, and the experiment was strictly conducted in accordance with the approved guidelines of the committee. Informed written consent was obtained from participants after procedural details had been explained.

### Subjects

Five participants aged 22–44 years old, three males and two females, participated in a color-matching experiment. Participants aged 22–44 years old, participated in a validation test. Two out of whom participated in both experiments. All participants had normal vision or corrected normal vision with glasses or contact lenses. The color vision was confirmed to be trichromatic by the Ishihara Plate.

### Apparatus

Stimuli were presented on a liquid crystal monitor (UP2516D, Dell; 2560 × 1440 pixels, 59 Hz frame rate, Adobe RGB). The monitor was connected via a HDMI cable to the onboard graphics card (Intel HD Graphics 620, 8 bits) of a PC running Microsoft Windows 10 Home Edition (64 bits). Color and luminance were calibrated with the chroma meter CS-200 (Konica Minolta, Japan) under the same illumination condition as the experiment. Pixel intensity was measured in sub-pixel resolution using a microscope (Dinolite basic, Dinolite), and the confirmed gray lines were the same across stimuli. The experiment was controlled using custom software developed using MATLAB 2018a (Mathworks) and Psychtoolbox 3.0.15^35^.

### Stimulus

Stimuli were defined by CIE 1931 color space. For the purpose of comparison with previous studies, we calculated corresponding color in MacLeod–Boynton chromaticity coordinates^36,37^. The MacLeod–Boynton chromaticity coordinates are based on the Smith and Pokorny cone fundamentals^37^, and spectral intensity of the display was measured by an illuminance spectrophotometer CL-500A (Konica Minolta, Japan). There are two axes *l* and *s*, which are the relative activation of L- and M-cone (*l* = L/(L + M)), and the activation of S-cone (*s* = S/(L + M)) in an equiluminant plane, respectively. The unit of *s* is arbitrary and normalized here to 1.0 for equal-energy white.

The sample and comparison were presented on a gray background (CIE *x* = 0.312, *y* = 0.329, 51.5 cd/m^2^, *l* = 0.65, *s* = 0.85) side-by-side at 1.24° apart as showed in Fig. 3, and their position on the left or right were randomized. A gray vertical line (1 pixel in width, 0.03°, *test line*) was placed in the center of the colored square (101 × 101 pixel in width 3.12°, *inducer area*). There were three conditions of the sample stimuli: *white-contour condition* with a white contour (Fig. 2B), *black-contour condition* with a black contour (Fig. 2C), and *no-contour condition* without any flanking contours (Fig. 2D). A white contour (1 pixel in width, 0.03°, CIE *x* = 0.312, *y* = 0.330, 210.2 cd/m^2^, *l* = 0.65, *s* = 0.85) or a black contour (1 pixel in width, 0.03°, CIE *x* = 0.312, *y* = 0.330, 8.5 cd/m^2^, *l* = 0.65, *s* = 0.85) was placed adjacent to the left and right edge of the test line. The test line was gray (CIE *x* = 0.313, *y* = 0.330, *l* = 0.65, *s* = 0.85) and had three levels of luminance (50.0, 75.0, 150.0 cd/m^2^). The inducer area was either cyan (CIE *x* = 0.278, *y* = 0.330, 189.0 cd/m^2^, *l* = 0.63, *s* = 0.92) or red (CIE *x* = 0.340, *y* = 0.330, 190.1 cd/m^2^, *l* = 0.66, *s* = 0.79). These two colors differed only in the luminance of the red and cyan (i.e., green + blue) pixels of the display. The comparison stimulus had a white square (101 × 101 pixel, 3.12°), which was the same color and luminance as the white contour, and one vertical line (1 pixel in width, 0.03°) located at the center.

### Procedure

The head of the participants was secured on a chin rest with a viewing distance of 40 cm from the monitor. The participants were asked to adjust the color and luminance of the line of the comparison to match the appearance of the line of the sample, using a trackball. Vertical and horizontal manipulations of the trackball induced luminance and color changes, respectively. The initial color and luminance of the comparison's vertical line were randomized. Fixation was not required. After the participants were satisfied with the matching, they pressed the button to complete the trial and proceed to the next trial with a 300 ms blank screen (D65, 50 cd/m^2^).

Before the experiment, the participants were trained on the apparatus and performing symmetric color matching while adapting to the illuminated room. The training stimuli were composed of a sample and a comparison, both had a thin line (1 px in width 0.03°) on a white background. The thin line of the sample was either dark gray (CIE *x* = 0.313, *y* = 0.330, 50.0 cd/m^2^, *l* = 0.65, *s* = 0.85), red (CIE *x* = 0.340, *y* = 0.330, 190.1 cd/m^2^, *l* = 0.66, *s* = 0.79), or cyan (CIE *x* = 0.278, *y* = 0.330, 189.0 cd/m^2^, *l* = 0.63, *s* = 0.92). Each participant repeated the training 2 ~ 3 times until they became competent on the trackball's operation.

In the experiment, there were 18 sample stimuli, including two colors of the inducer, three luminance levels of the test line, and the white-contour, black-contour, and no-contour conditions. The sample stimuli were presented in random order. The time required for the matching of all presentation stimuli was about 15 min. Each participant repeated the matching three times with at least a 7-min break between sessions.

### Optical validation tests

In the longitudinal aberration test, a square annulus for the fixation and the computer monitor was orthogonally arranged, and those two images were superimposed by a half-transparent mirror angled at 45°. The depth of the annulus was adjustable, while the depth of the monitor was kept constant. The annulus (29.5 × 21.0 cm, 30.0 × 21.6° at 55 cm visual distance) had a dark aperture (3.0 × 3.0 cm, 3.1° × 3.1° at 55 cm visual distance) in the center. The stimulus image on the monitor was presented at the aperture location. The participants were instructed to report the color appearance of lines of the monitor while they gazed at the annulus. To help participants focus on the annulus, a fine texture was printed on the surface. The focusing depth was either + 1 (far), − 1 (near), or 0 (zero) cm relative to the depth of the monitor. The stimulus (312 × 312 pixels, 7.01°) was the vertical pale gray line (1 pixel, 0.02°) with the white contour on the colored background. The color of the background (inducer) was either cyan, red, or white.

In the lateral aberration test, the participants gazed at a fixation point at the center of the monitor, and the stimulus image was presented to the right visual field and subtended 0.99°–8.00°. Participants were instructed to report the color appearance of the lines. The stimuli (312 × 312 pixels, 7.01°) were pale gray lines with the white contour on the colored background. The gray lines were either vertical or horizontal, and the color of the background (inducer) was either cyan, red, or white. In this test, the stimulus contains seven equally spaced gray lines instead of single line. This repetition was intended to reduce the effect of eccentricity between vertical and horizontal stimuli.

Verbal responses of color appearance were classified into three categories: reddish (terms including red, pink, orange, or yellow), bluish (terms including blue, water, or cyan), and achromatic (gray, black). When both colored and achromatic terms were used simultaneously (i.e. bluish gray), the response was classified based on color (i.e., bluish). No response was observed when using both the reddish and bluish terms simultaneously. This classification was valid for all responses except for one response (green).

## Supplementary information

Supplementary information.
