# Supplementary material for: Large enhancement of simultaneous color contrast by white flanking contours
Source: Sci Rep. 2020 Nov 18;10:20136. doi: 10.1038/s41598-020-77241-5 (PMC7674406; doi:10.1038/s41598-020-77241-5)
Supplement: Supplementary file 1 — Supplementary information. [file 41598_2020_77241_MOESM1_ESM.docx]

## Supplemental figure for

## Large enhancement of simultaneous color contrast by white flanking contours

## Tama Kanematsu^1,2^, Kowa Koida^1,3,*^

^1^Department of Computer Science and Engineering, Toyohashi University of Technology, Aichi, 441-8580, JAPAN

^2^Research Fellow of Japan Society for the Promotion of Science

^3^Electronics-Inspired Interdisciplinary Research Institute (EIIRIS), Toyohashi University of Technology, Aichi, 441-8580, JAPAN

[*koida@tut.jp](mailto:*koida@tut.jp)

+81-532-44-1309

# Supplemental Figure S1.


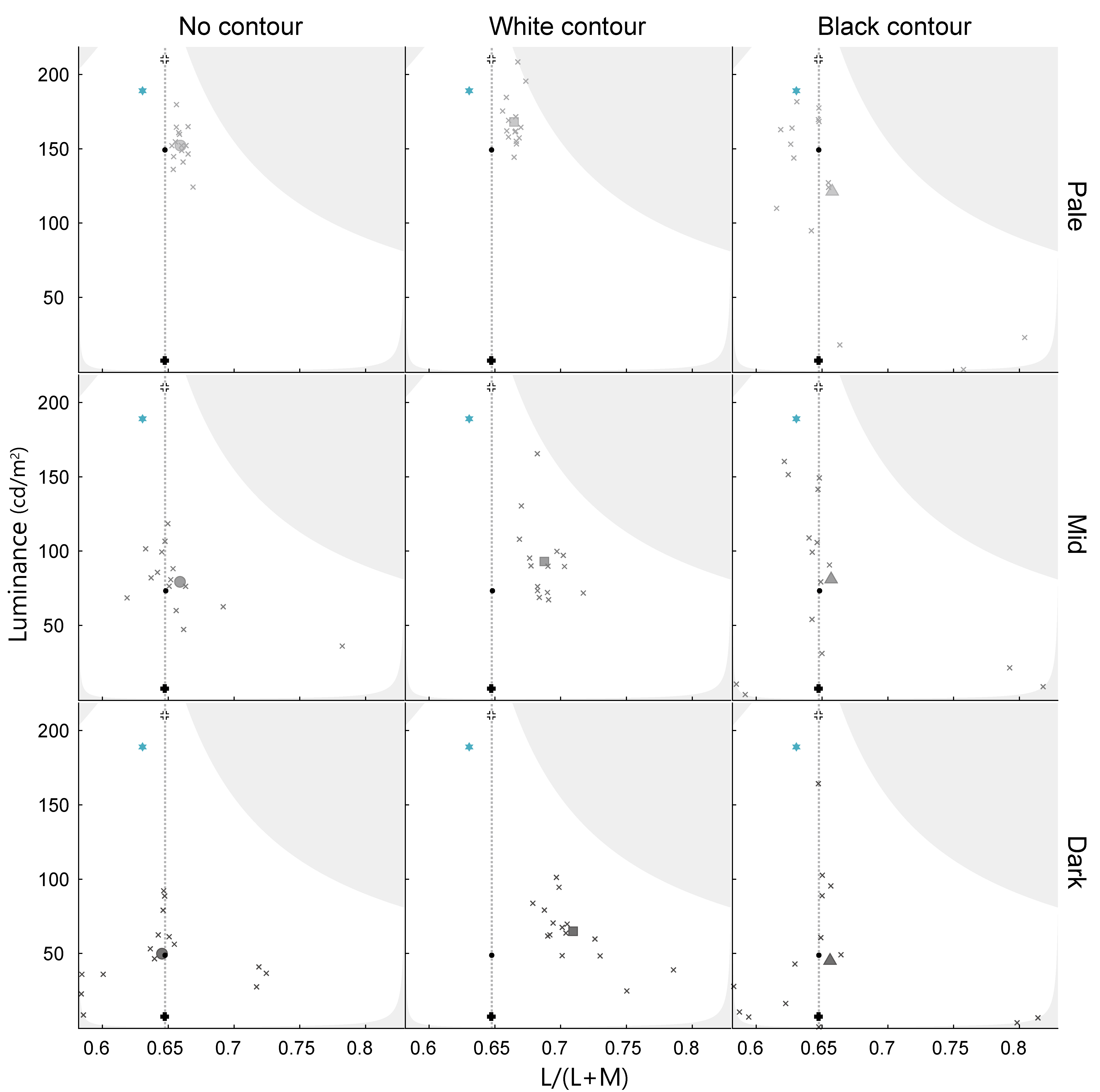


Individual results of the appearance matching of the cyan inducer (5 observers × 3 repetitions). Panels from left to right show the results of no-, white-, and black-contour conditions. Panels from top to bottom show the results of pale (150 cd/m^2^), mid (75 cd/m^2^), and dark (50 cd/m^2^) test lines. Axes are the same as in figure 5E, F in the main text. The small black dot on the vertical line (D65) represents the levels of the test lines of the sample. The colored star represents the inducer of the sample. Small crosses represent each matching. Circles, squares, and triangles represent the mean of the no-, white-, and black-contour conditions, respectively. White and black crosses represent the color of the white and black contours of the sample stimuli, respectively.

# Supplemental Figure S2.


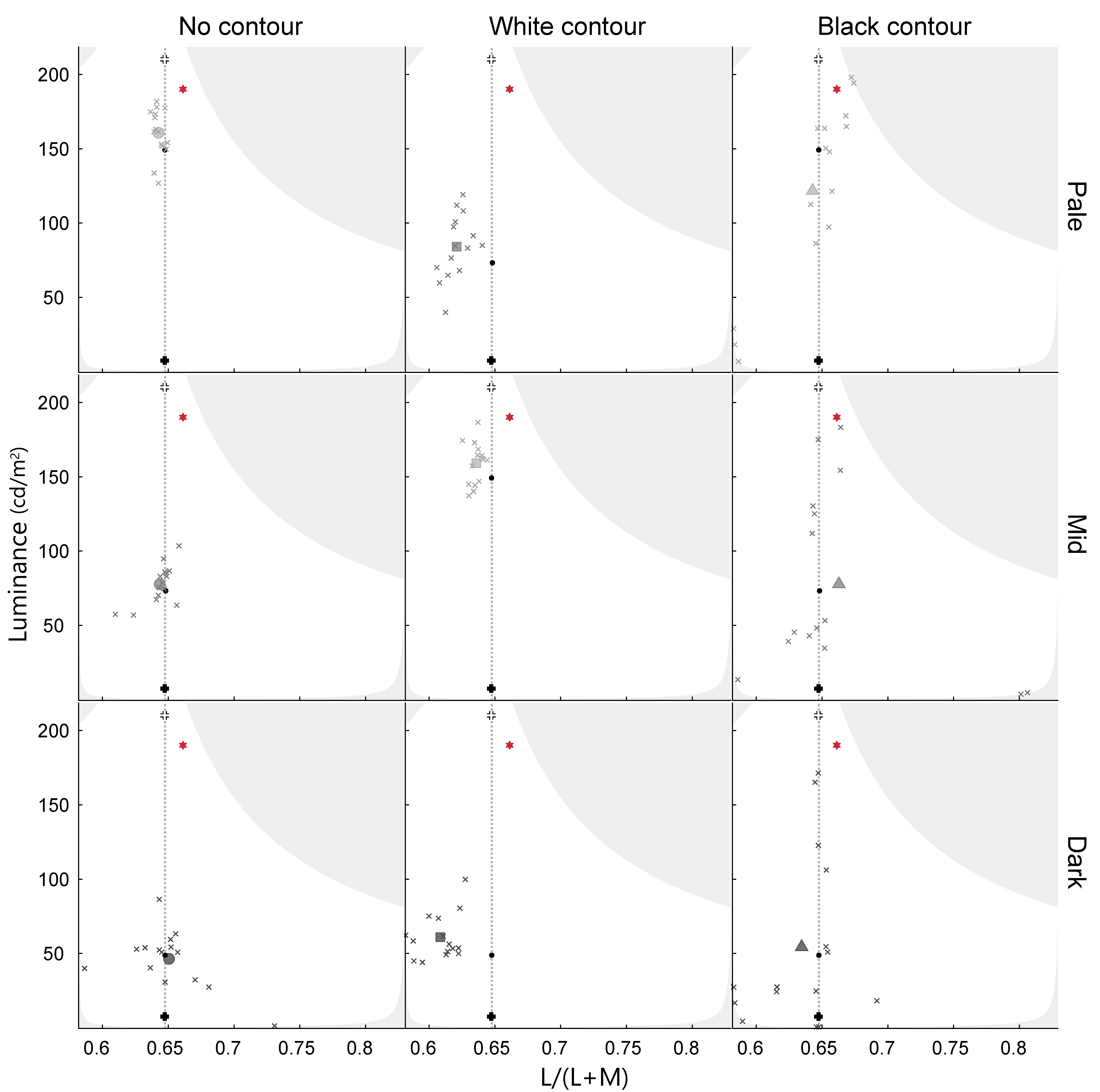


Individual results of the appearance matching of the red inducer. The format is the same as Fig. S1.
